# The Possible Role of Complete Loss of Myostatin in Limiting Excessive Proliferation of Muscle Cells (C2C12) via Activation of MicroRNAs

**DOI:** 10.3390/ijms20030643

**Published:** 2019-02-02

**Authors:** Peixuan Huang, Daxin Pang, Kankan Wang, Aishi Xu, Chaogang Yao, Mengjing Li, Wenni You, Qiushuang Wang, Hao Yu

**Affiliations:** 1 Jilin Provincial Key Laboratory of Animal Embryo Engineering, College of Animal Sciences, Jilin University, Changchun 130062, China; huangpx16@mails.jlu.edu.cn (P.H.); pdx@jlu.edu.cn (D.P.); wang4196@purdue.edu (K.W.); xuas14@mails.jlu.edu.cn (A.X.); yaocg13@mails.jlu.edu.cn (C.Y.); mjli18@mails.jlu.edu.cn (M.L.); youwn18@mails.jlu.edu.cn (W.Y.); 2 Public Computer Education and Research Center, Jilin University, Changchun 130062, China

**Keywords:** MSTN, RNA-seq, miRNA-seq, transcriptomics, C2C12, RMS

## Abstract

Myostatin (MSTN) is a member of the TGF-β superfamily that negatively regulates skeletal muscle growth and differentiation. However, the mechanism by which complete MSTN deletion limits excessive proliferation of muscle cells remains unclear. In this study, we knocked out MSTN in mouse myoblast lines using a Clustered Regularly Interspaced Short Palindromic Repeats (CRISPR/Cas9) system and sequenced the mRNA and miRNA transcriptomes. The results show that complete loss of MSTN upregulates seven miRNAs targeting an interaction network composed of 28 downregulated genes, including *TGFB1*, *FOS* and *RB1*. These genes are closely associated with tumorigenesis and cell proliferation. Our study suggests that complete loss of MSTN may limit excessive cell proliferation via activation of miRNAs. These data will contribute to the treatment of rhabdomyosarcoma (RMS).

## 1. Introduction

Myostatin (MSTN) [1], also known as growth/differentiation factor-8 (GDF-8), is a member of the TGF-β superfamily. In previous studies, MSTN has been confirmed to be a secreted growth factor expressed predominantly in skeletal muscle [2,3] and to negatively regulate myoblast growth and differentiation [4]. Moreover, MSTN also controls the activation and proliferation of satellite cells, the stem cells of skeletal muscle [5]. MSTN was first discovered in mice [1], but was later found to be highly conserved across species. Homozygous disruption of the *MSTN* gene [1] or naturally occurring *MSTN* gene mutations, e.g., in humans [6], mice [7], cattle [8] and sheep [9], result in widespread increases in skeletal muscle mass (the “double-muscled” phenotype) [10,11].

In contrast, rhabdomyosarcoma (RMS) [12] is a heterogeneous tumor that has been confirmed to develop as a result of genetic alterations in mesenchymal progenitor/stem cells, which express some markers of normal skeletal muscle but show an ability to proliferate indefinitely and do not completely differentiate into the muscle phenotype [13]. A previous study showed that MSTN is expressed and translated in the cultured RMS cell line originally derived from embryonic RMS cell lines, RD. The addition of exogenous recombinant MSTN inhibits the proliferation of RD cells cultured in growth media, consistent with the role of MSTN in normal myoblast proliferation inhibition [14]. However, the muscle development in *MSTN* mutation animals remains limited [15,16] whether the mutation is caused by natural mutations [8,11,17] or artificial induction [18], whereas the muscle of animals with RMS will exhibit indeterminate growth. MicroRNAs (miRNAs or miRs) are endogenous ~22-nt RNAs that can play critical roles in gene regulation by pairing to the messages of protein-coding genes to specify mRNA cleavage and suppress gene expression, resulting in the repression of productive translation or mRNA decay [19,20]. miRNAs have been implicated in many biological processes, such as tumorigenesis [21], stem cell differentiation [22] and organ development [23]. Recent research has shown that miRNAs also affect the proliferation and differentiation of skeletal myoblasts by interacting with MSTN [24,25]. Therefore, we suspected that the complete absence of the most important negative regulator of skeletal muscle development and growth, MSTN, would activate some miRNAs. Several of these miRNAs would target some genes that regulated the growth and development of myoblasts and trigger a negative feedback mechanism to suppress excessive skeletal muscle proliferation.

Hence, in this study, we knocked out the *MSTN* gene in mouse myoblasts using CRISPR/Cas9 [26] and acquired the sequencing data through RNA-seq and miRNA-seq with transcriptome data for RMS. We aimed to explore the potential relationship between miRNAs and muscle overgrowth resulting from the loss of *MSTN* by comparing the sequencing data between MSTN-knockout (KO) and RMS. Our findings suggest that *MSTN* may be a very promising therapeutic target for the treatment of myosarcoma caused by abnormalities in skeletal muscle development.

## 2. Results

### 2.1. Generation of MSTN-Knockout (KO) Cell Lines

**Figure 1:**
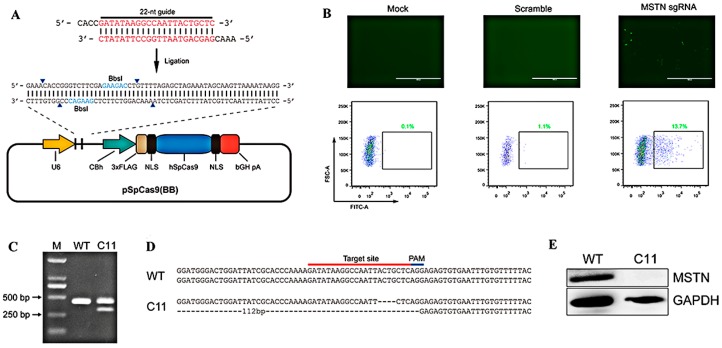
Preparation and validation of knockout (KO) cells. (**A**) Schematic of the recombinant plasmid. The plasmid was designed with the enzyme restriction sites after the U6 promoter. (**B**) Fluorescence microscopy images of cells. From left to right: mock group, scramble group and myostatin (MSTN) sgRNA group (scale bar = 1000 μm). (**C**) Electrophoretogram of the results of cell validation by PCR. (**D**) Wild-type (WT) and C11 sequences. C11 cells have a deleted section due to the sgRNA. (**E**) Identification of MSTN protein levels in WT and C11 cells by Western blot analysis. There was no MSTN protein expression in C11 cells.

We first selected exon 3 of *MSTN* as a potential target site for sgRNAs and designed a BbsI restriction site behind the U6 promoter. We then set a 3× FLAG tag ahead of a nuclear localization sequence (NLS) and *Streptococcus pyogenes* Cas9 nickase (nSpCas9) to observe the fluorescence of cells (Figure 1A). After the synthetic plasmid was transfected into C2C12 cells through electroporation, the cells containing *MSTN* sgRNA were identified with green fluorescence under a fluorescence microscope (Figure 1B). Upon agarose gel electrophoresis, wild-type (WT) cells demonstrated a 480-bp band for the *MSTN* PCR product, while the KO cells (C11) had two bands at 480 and 300 bp (Figure 1C). In addition, the sequencing data of genomic DNA extracted from WT cells and C11 cells confirmed that the target sequence of *MSTN* was effectively destroyed in C11 cells (Figure 1D). At the protein level, C11 cells showed a lack of MSTN protein compared to WT cells in Western blot analysis (Figure 1E).

### 2.2. Identification of Differentially Expressed Genes (DEGs) and Differentially Expressed miRNAs (DEMs) in MSTN-KO Cells

**Figure 2:**
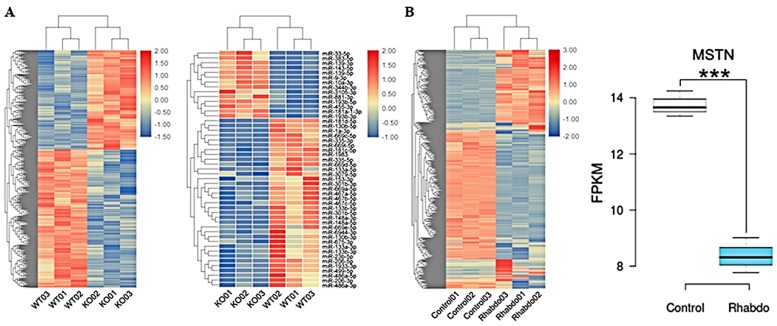
Differentially expressed genes (DEGs) and differentially expressed miRNAs (DEMs) in KO cell lines. (**A**) Heatmap hierarchical clustering revealed the presence of DEGs and DEMs in the MSTN-KO groups compared with the control groups. (**B**) Expression of DEGs displayed by heatmap hierarchical clustering and the expression levels of MSTN in the control groups and the rhabdo groups. *** *p* < 0.001.

We analyzed four types of sample groups in our experiments, including KO, wild-type (WT), RMS (rhabdo) and control groups, with three replicates of each group. Differentially expressed genes (DEGs) and differentially expressed miRNAs (DEMs) were obtained based on fragments per kilobase of exon per million mapped (FPKM) values representing the gene expression levels. A total of 1236 DEGs and 62 DEMs were obtained for the KO groups compared to the WT groups, including 721 upregulated DEGs, 515 downregulated DEGs, 24 upregulated DEMs and 38 downregulated DEMs, as shown in the hierarchical clustering heatmaps (Figure 2A). Compared to the control groups, the rhabdo groups had 682 upregulated genes and 827 downregulated genes. In addition, the expression of MSTN in the rhabdo group was significantly reduced from 13.94 to 8.36 compared to the control group, as shown in Figure 2B.

### 2.3. Identification of miRNA-Influenced DEGs

**Figure 3:**
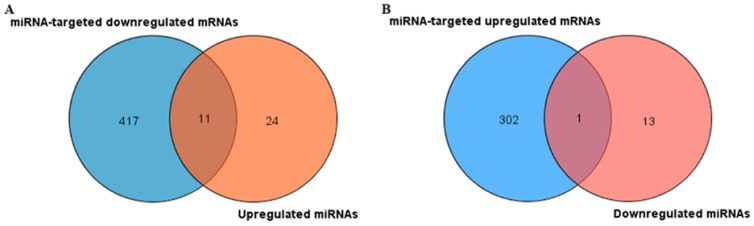
Identification of miRNAs involved in mRNA regulation. (**A**) Thirty-five upregulated miRNAs were identified, 11 of which led to the downregulation of target DEGs. (**B**) Fourteen downregulated miRNAs were identified, only one of which led to the upregulation of target DEGs.

In total, 35 upregulated DEMs and 14 downregulated DEMs were screened under the evaluated criteria of gene differential expression level (*p* ≤ 0.05 and ≥2-fold change). We further predicted that 11 upregulated miRNA and one downregulated miRNA have targeted genes in DEGs through the NetworkAnalyst web server, as shown in Figure 3. All extensively overlapping DEMs are displayed in Table 1.

### 2.4. Functional Enrichment Analysis

**Figure 4:**
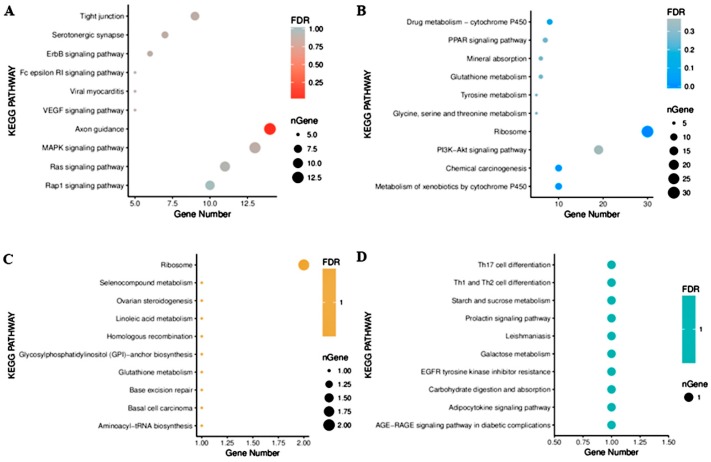
Functional enrichment analysis of DEGs and DEMs. (**A**) Significantly enriched pathways for the upregulated DEGs were ranked by *p*-values. (**B**) Pathways in which the downregulated DEGs were significantly involved. (**C**,**D**) Pathways in which the upregulated or downregulated DEMs were significantly enriched.

To explore the pathways associated with the DEGs, MSTN-KO cell lines and rhabdo cell lines were subjected to enrichment analysis for KEGG pathways. In the MSTN-KO group, the most significant pathway among the top 10 enriched pathways for upregulated genes was axon guidance (Figure 4A), whereas the ribosome pathway was the most significant pathway among the top 10 enriched pathways in downregulated genes. Furthermore, we found 20 genes enriched in the PI3K-Akt pathway (Figure 4B), the inhibition of which enhances sensitivity to fas-mediated apoptosis in a human carcinoma cell line [27]. Inexplicably, the DEGs of the rhabdo group were not significantly enriched in any pathways and only one or two genes were enriched in each of the top 10 pathways (Figure 4C,D).

### 2.5. Differential Expression of Apoptotic Genes between the MSTN-KO and Rhabdo Groups

**Figure 5:**
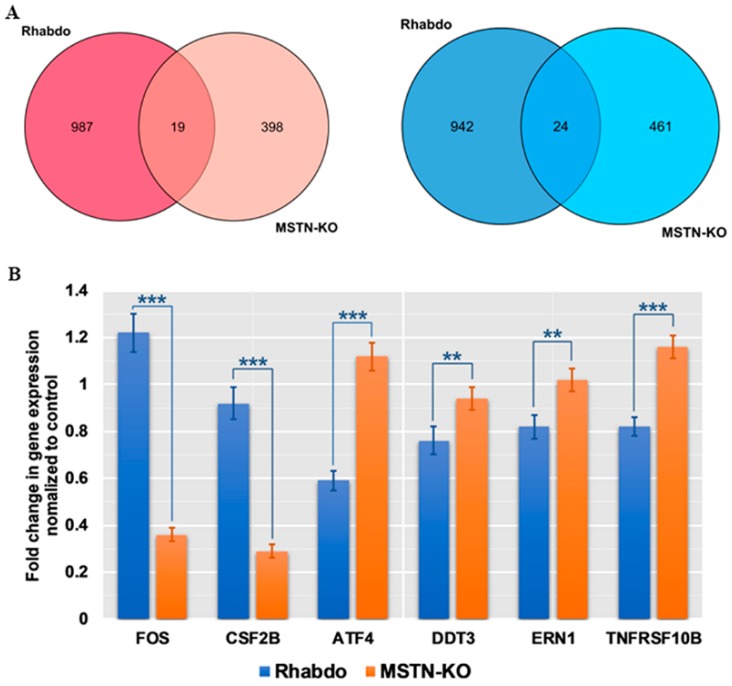
Expression analysis of DEGs between the MSTN-KO and rhabdo groups. (**A**) Overlapping DEGs between the MSTN-KO and rhabdo groups. (**B**) Apoptosis-related gene expression differences between the KO and rhabdo groups. All data are shown as the mean ± SEM. Two-tailed Mann–Whitney *U* tests were used for pairwise comparisons between groups. ** *p* < 0.01, *** *p* < 0.001.

Upon overlapping the DEGs of the rhabdo and MSTN-KO groups, we found that only 19 upregulated genes and 24 downregulated genes were shared in both groups (Figure 5A). Next, we compared the apoptotic DEGs in the MSTN-KO and rhabdo groups. We found that two proapoptotic genes (*ATF4* and *TNFRSF10B*) were upregulated and two antiapoptotic genes (*FOS* and *CSF2B*) were downregulated in the MSTN-KO group compared to the rhabdo group. However, only one proapoptotic gene (*FOS*) was upregulated and three antiapoptotic genes (*ATF4*, *DDT3* and *ERN1*) were downregulated in the rhabdo group compared to the MSTN-KO group, as shown in Figure 5B.

### 2.6. Interactive miRNA–mRNA Network

**Figure 6:**
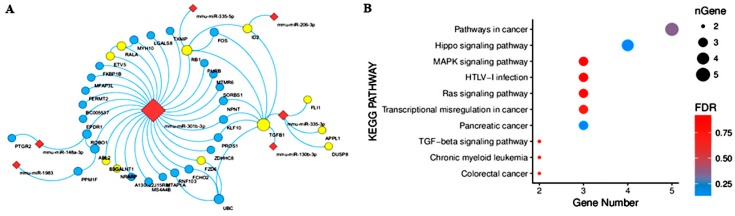
miRNA–mRNA network in the MSTN-KO group. (**A**) Interactive network of upregulated miRNAs and downregulated mRNAs in the MSTN-KO group. (**B**) KEGG pathway enrichment of DEGs in the miRNA–mRNA network.

A network was constructed with the STRING database to explore associations between miRNAs and DEGs. It was displayed with Cytoscape 3.6.1. We found that seven miRNAs and 37 DEGs formed an interaction network. In this network, mmu-miR-301b-3p targeted most (28) of the DEGs. *TGFB1*, *RB1* and *EPDR1* were regulated by two miRNAs, and *TGFB1* and *RB1* were coexpression factors of the *FOS* gene. KEGG enrichment analysis revealed that 11 genes (the nodes marked yellow in the network) were involved in tumor formation, tumor development and mismatch repair, as shown in Figure 6A,B.

### 2.7. Identification and Validation of the Selected Genes and miRNAs

**Figure 7:**
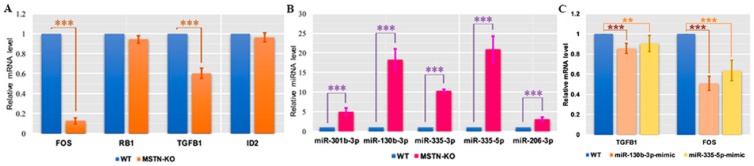
Expression of selected genes and miRNAs in the WT and MSTN-KO groups. (**A**,**B**) Expression levels of four DEGs and five DEMs in the WT and MSTN-KO groups measured by qRT-PCR and normalized to GAPDH and U6 snRNA expression levels, respectively. (**C**) Expression levels of *TGFB1* and *FOS* in WT, miR-130b-3p mimics and miR-335-5p groups. The experiments were performed in triplicate with three biological replicates for each gene or miRNA. All data are shown as the mean ± SEM. Two-tailed Mann–Whitney U tests were used for pairwise comparisons between groups. ** *p* < 0.01, *** *p* < 0.001.

To verify the RNA-seq and miRNA-seq results, four mRNAs (*FOS*, *RB1*, *TGFB1* and *ID2*) and five miRNAs were selected and tested by qRT-PCR. We found that the expression levels of only *FOS* and *TGFB1* were significantly downregulated in the MSTN-KO group compared with the WT group (Figure 7A) which is consistent with the RNA-seq results. In contrast, the expression levels of five miRNAs (miR-301-3p, miR-130b-3p, miR-335-3p, miR-335-5p and miR-206-3P) were all significantly upregulated in the MSTN-KO group compared with the WT group, as shown in Figure 7B. Among them, the expression levels of miR-130b-3p and miR-335-5p increased the most obviously.

To further confirm the regulation of miR-130b-3p and miR-335-5p by the expression of *TGFB1* and *FOS*, we detected the mRNA expression levels of *TGFB1* and *FOS* in C2C12 cells transfected with the mimics of miR-130b-3p and miR-335-5p, respectively, under full medium conditions by qRT-PCR. We found both miRNAs could downregulate the mRNA levels of *TGFB1* and *FOS*; however, the *FOS* gene seems to be more affected by negative regulation from miRNA mimics than *TGFB1*. The expression trends of the two genes were consistent with those in the MSTN-KO cell line (Figure 7C).

## 3. Discussion

MSTN is a secreted growth factor of the TGF-β superfamily, expressed predominantly in skeletal muscle, that inhibits the growth and development of muscle. A lack of MSTN may result in a “double-muscled” phenotype. However, low expression or absence of MSTN leads to neither excessive muscle growth nor myosarcoma, which involves unrestricted muscle development. Previous studies have shown that *MSTN* is regulated by several miRNAs, such as miRNA-218 [25,28] and miR-27 [24,29]. Currently, the pathogenic mechanism of myosarcoma remains unclear and there is also a lack of effective therapeutic strategies for myosarcoma [30]. In our study, we discovered that *MSTN* expression was decreased with RMS overgrowth. Therefore, we sought to discover miRNAs in skeletal muscle cells that can be used to treat RMS proliferation using an MSTN-KO cell line. This was a trial study that may provide some useful suggestions for the treatment of myosarcoma.

Notably, the transcriptome patterns of the RMS group and the MSTN-KO group differed greatly and few upregulated and downregulated genes were shared between the two groups. The DEGs of the rhabdo group were not appreciably enriched in any pathway, indicating that it is not feasible to use pathway enrichment methods to find pathways for RMS activation and that cell proliferation and apoptosis in skeletal muscle are distinctly different from those in other tissues. In the MSTN-KO group *MSTN* was destroyed and this complete deletion of *MSTN* changed miRNA transcription, upregulating several miRNAs but downregulating only one miRNA. Therefore, we suspected there may be some other factors that also contribute to the change of gene expression. Our miRNA–mRNA targeting results showed that most of the downregulated mRNAs targeted by upregulated miRNAs were enriched in tumor pathways. However, the MSTN-KO cells would not form myosarcomas, but undergo normal apoptosis. The q-PCR results showed that the *TGFB1* and *FOS* genes were significantly decreased while respectively overexpressed miR-130b-3p and miR-335-5p in MSTN-KO cell lines. In addition, a previous study confirmed that the presence of an miR-335 mimic decreased the transcript expression levels of *TGFB1*, while the miR-335 inhibitor increased it in gastric cancer cell lines [31]. In general, our results showed that miR-130b-3p and miR-335-5p were significantly overexpressed in MSTN-KO cell lines and may restrict the excessive proliferation of skeletal muscle cells by suppressing the mRNA activity of *TGFB1* and *FOS*.

As the interaction network of upregulated and downregulated miRNAs displays, we found that *FOS*, *TGFB1*, *RB1* and *ID2* are important genes controlling proliferation and apoptosis in myosarcoma. Only *FOS* and *TGFB1* were significantly downregulated, as validated by q-PCR. According to the comparison of differentially expressed apoptotic genes between the RMS group and the MSTN-KO group, we found that the *FOS* gene was significantly downregulated in the MSTN-KO group, which was helpful in maintaining normal cell apoptosis [32,33]. *FOS*, also known as *c-fos* in previous studies, a proto-oncogene, is a negative regulator of MyoD expression and myoblast differentiation [34,35]. Overexpression of *c-fos* would inhibit myogenic differentiation [36] and the downregulation of *c-fos* is associated with the differentiation of myoblasts in culture triggered by the withdrawal of mitogens during myogenesis [37]. In addition, *c-fos* is also expressed during early bone differentiation [38,39] and regulates endochondral osteogenesis in bone formation and fracture healing [40,41]. Overexpression of *c-fos* plays an essential role during bone tumor formation [42,43]. In transgenic mouse models, the induction of *c-fos* expression using a ubiquitous promoter causes the development of bone tumors with 100% penetrance [38,44]. Our results show that the overexpression of miR-130b-3p and miR-335-5p indeed downregulate the *FOS* gene, so we hypothesized that the deletion of MSTN would indirectly maintain cell apoptosis and inhibit tumor proliferation through miR-130b-3p and miR-335-5p, although *FOS* was not directly silenced by the DEMs.

In contrast, *TGFB1* was simultaneously regulated by two miRNAs, which were the two most upregulated miRNAs, as verified by q-PCR. Another study indicated that cells treated with *TGFB1* [45,46] undergo the epithelial-to-mesenchymal transition (EMT), which is characterized by a loss of cell–cell contacts, the emergence of spindle-shaped fibroblast-like mesenchymal cells and the induction of the expression of mesenchymal cell markers, such as N-cadherin, fibronectin and vimentin [47]. However, EMT is one such multifaceted molecular program in malignant transformation causing loss of epithelial characteristics and gain in the mesenchymal phenotype [48]. EMT is believed to be a vital mechanism for carcinoma progression and is associated with aggressive behavior of cancer cells [49]. In particular, deregulating the *TGFB1* signaling would involve both the development of RMS and the loss of cell–cell adhesion, leading to aberrant cell migration and deregulated growth [50,51]. Moreover, *TGFB1* signaling is essential for articular cartilage homeostasis. *TGFB1* is rapidly upregulated by mechanical loading and it stimulates proteoglycan synthesis and blocks the expression of chondrocyte hypertrophic genes [52]. However, *TGFB1* signaling is deregulated in osteoarthritis and high concentrations of *TGFB1* can be found in the synovial fluids of patients with this disease [53]. High levels of *TGFB1* result in the preferential activation of the SMAD1/5/8 pathways instead of the SMAD2/3 pathways and cause the upregulation of genes involved in fibrogenesis and hypertrophy, leading to synovial fibrosis and osteophyte formation [54]. What puzzled us is that although *TGFB1* was targeted by miRNAs, the overexpression of miRNA mimics did not cause a substantial decrease of *TGFB1*. Therefore, we speculate that miRNA’s regulatory role on *TGFB1* in muscle cells is only to limit the over-proliferation of myoblasts and prevent the occurrence of tumors.

In our study, we elucidated a potential pathway of miRNA activation induced by the complete loss of MSTN. The activated miRNAs may inhibit excessive proliferation and promote apoptosis in skeletal muscle cells and suppress some tumorigenic genes. These data may greatly contribute to the development of RMS treatments utilizing these candidate miRNAs.

## 4. Materials and Methods

### 4.1. Preparation of Cells and Cell Culture

In this study, we selected mouse myoblasts (C2C12 cells), which were preserved in our laboratory (Jilin university, Changchun, China), as the experimental cells. The C2C12 cells were cultivated in basal medium containing Dulbecco’s modified Eagle’s medium (DMEM, Gibco, Grand Island, New York, USA) with 10% fetal bovine serum.

### 4.2. Construction of Plasmids and KO of MSTN

We selected the plasmid px330 as the vector backbone for this research and ligated the sgRNA we designed into the plasmid. Two complementary sgRNA oligo DNAs were combined, annealed to form double-stranded DNA in the presence of 10× NEB standard Taq buffer and cloned into the BbsI sites of the px330 plasmid to form the intact targeting vector. The MSTN gene fragment was knocked out in cells by electrotransfection. Approximately 3 × 106 PFFs were floated in 250 µL of Opti-MEM (Gibco, Grand Island, New York, USA) and electroporated using BTX ECM 2001 in 2-mm gap cuvettes. The parameters for electroporation were as follows: 300 V, 1 ms, three pulses for one repeat, as previous study described [55]. In different electroporation experiments, 25 µg of the corresponding plasmids (with or without 3 µM oligonucleotides) was used. The cells were assessed by fluorescence microscopy (Olympus BX51, Olympus, Tokyo, Japan) under appropriate excitation filters after observation at 24 h post-electroporation. Cells exhibiting the green fluorescent label were collected using trypsin digestion. The harvested cells were washed twice, then resuspended with 300 µL of PBS and analyzed using a BD Accuri C6 flow cytometer (BD, Franklin Lakes, NJ, USA).

### 4.3. Data Collection

RNA was extracted from two groups of C2C12 cell lines (the KO group and the WT group) using standard chloroform extraction. Subsequent sequencing of a total of six samples (three replicates of the KO group and three replicates of the WT group) was performed on an Illumina HiSeq 2500 system (Illumina, San Diego, CA, USA). Raw and processed RNA-seq data and miRNA-seq data were deposited at GSE124195 and GSE124194. In addition, the sample data of RMS were obtained from the GSE29775 dataset from NCBI’s GEO database. The control group and RMS group contain three repetitions each.

### 4.4. Differentially Expressed Gene (DEG) Analysis

We acquired expression information from RNA-seq data and miRNA-seq data for both the KO and WT cell groups based on the fragments per kilobase of exon per million fragments mapped (FPKM) values. To identify DEGs and DEMs, we used iDEP software (http://bioinformatics.sdstate.edu/idep/) and set the cutoff criteria as a *p*-value < 0.05 and a logFC >1 or <−1. Then, we used TBtools 0.6654 [56] to identify up- or downregulated DEMs overlapping with down- or upregulated DEGs, respectively.

### 4.5. Gene Enrichment Analysis

The online platform WebGestalt [57] was used to perform gene pathway enrichment analysis. All obtained DEGs were submitted as a list. We analyzed the Kyoto Encyclopedia of Genes and Genomes (KEGG) pathways of significant terms to observe the enriched pathways for the DEGs. A *p*-value < 0.05 was considered statistically significant in screening the enrichment results. In addition, all DEMs were also submitted to WebGestalt to determine their enriched KEGG pathways with a cutoff *p*-value of <0.05.

### 4.6. Construction of a miRNA–mRNA Interactive Network

The Search Tool for the Retrieval of Interacting Genes/Proteins (STRING) database (https://string-db.org/cgi/input.pl) was used to construct protein–protein interaction (PPI) networks of DEGs in the MSTN-KO group. Prediction of multiple miRNA targets was performed with the online program NetworkAnalyst [58]. The miRNA–mRNA network was analyzed and visualized with Cytoscape 3.6.1.

### 4.7. Identification and Validation of mRNAs and miRNAs by qRT-PCR

To confirm our results, qRT-PCR analyses were conducted with the selected mRNAs and miRNAs likely to play important roles in the interaction network. RNA was carefully isolated from cells using TRIzol reagent and 2 micrograms of total RNA was reverse transcribed using a Quantscript RT Kit (Tiangen, Beijing, China). The resulting first strand cDNA was diluted 5-fold and used as the template for qRT-PCR analysis with an iCycler iQ Real-Time PCR Detection System (BIO-RAD Laboratories, Richmond, CA, USA). GAPDH and U6 snRNA served as the reference genes in the mRNA and miRNA groups, respectively. The primer sequences designed for the target mRNAs and miRNAs are listed in Table 2.

To further explore whether the *FOS* and *TGFB1* genes were downregulated by miR-130b-3p and miR-335-5p, we attempted to overexpress the two miRNA in WT cell lines and detect the expression levels of *FOS* and *TGFB1* genes. Lipofectamine 2000 reagent (Invitrogen, Life Technologies, Carlsbad, CA, USA) was used to transiently transfect the miR-130b-3p and miR-335-5p mimics (Ruibo, Guangzhou, China) into C2C12 cells as per the manufacturer’s instructions. Briefly, cells were seeded in a 6-well plate with 2 × 105 cells per well and transfected with 30 nM of mimics of miR-130b-3p (5′-CAGTGCAATGATGAAAGGGCAT-3′) and miR-335-5p (5′-TCAAGAGCAATAACGAAAAATGT-3′) (New human and mouse microRNA genes found by homology search) until 70% confluence was reached. Thirty nmol of a random sequence of miRNA molecules were used as negative controls. After transfection, cells were added to pre-warmed media and immediately placed in an incubator at 37 °C in a 5% CO_2_ atmosphere. Cells were harvested at 48 h after transfection for functional assays.

## Figures and Tables

**Table 1:** Overlapping miRNAs regulating target DEGs.

Group of miRNAs	Overlapping miRNAs	
Upregulated	mmu-miR-148a-3p	mmu-miR-130b-3p
	mmu-miR-133a-3p	mmu-miR-1933-3p
	mmu-miR-301b-3p	mmu-miR-335-5p
	mmu-miR-675-3p	mmu-miR-1a-3p
	mmu-miR-1983	mmu-miR-335-3p
	mmu-miR-206-3p	
Downregulated	mmu-miR-139-5p	

**Table 2:** Primers for the target mRNAs and miRNAs.

*mRNA*/*miRNA*	Forward Primer	Reverse Primer
*FOS*	CGGGTTTCAACGCCGACTA	TGGCACTAGAGACGGACAGAT
*TGFB1*	CCACCTGCAAGACCATCGAC	CTGGCGAGCCTTAGTTTGGAC
*RB1*	TCGATACCAGTACCAAGGTTGA	ACACGTCCGTTCTAATTTGCTG
*ID2*	TCCGGTGAGGTCCGTTAGG	CAGACTCATCGGGTCGTCC
*GAPDH*	AGGTCGGTGTGAACGGATTTG	GGGGTCGTTGATGGCAACA
*miR-301b-3p*	CAGCAGTGCAATAGTATTGTCA	AGGTCCAGTTTTTTTTTTTTTTTCAA
*miR-130b-3p*	CAGCAGTGCAATGATGAAAG	CAGGTCCAGTTTTTTTTTTTTTTTATG
*miR-335-3p*	CGCAGTTTTTCATTATTGCTCCT	GGTCCAGTTTTTTTTTTTTTTTGGT
*miR-335-5p*	GCAGTCAAGAGCAATAACGA	CAGGTCCAGTTTTTTTTTTTTTTTACA
*mir-206-3p*	GCAGTGGAATGTAAGGAAGT	CCAGTTTTTTTTTTTTTTTCCACACA
*U6 snRNA*	CACCACGTTTATACGCCGGTG	CACCACGTTTATACGCCGGTG

## Abbreviations

MSTN	myostatin
RMS	rhabdomyosarcoma
MSTN-KO	MSTN-knockout
DEGs	differentially expressed genes
DEMs	differentially expressed miRNAs
KEGG	Kyoto Encyclopedia of Genes and Genomes
WT	wild-type
FPKM	fragments per kilobase of exon per million fragments mapped
STRING	Search Tool for the Retrieval of Interacting Genes/Proteins
PPI	protein–protein interaction
